# Diffusion tensor imaging in elderly patients with idiopathic normal pressure hydrocephalus or Parkinson’s disease: diagnosis of gait abnormalities

**DOI:** 10.1186/2045-8118-9-20

**Published:** 2012-09-18

**Authors:** Kohei Marumoto, Tetsuo Koyama, Masashi Hosomi, Norihiko Kodama, Hiroji Miyake, Kazuhisa Domen

**Affiliations:** 1 Department of Physical Medicine and Rehabilitation, Hyogo College of Medicine, 1-1 Mukogawa-cho, Nishinomiya, Hyogo 663-8501, Japan; 2 Department of Rehabilitation Medicine, Nishinomiya Kyoritsu Neurosurgical Hospital, 11-1 Imazu-Yamanaka-cho, Nishinomiya, Hyogo 663-8211, Japan; 3 Department of Neurosurgery, Nishinomiya Kyoritsu Neurosurgical Hospital, 11-1 Imazu-Yamanaka-cho, Nishinomiya, Hyogo 663-8211, Japan

**Keywords:** Diffusion tensor imaging, Fractional anisotropy, Idiopathic normal pressure hydrocephalus, Parkinson’s disease, White matter

## Abstract

**Background:**

Gait abnormalities in the elderly, characterized by short steps and frozen gait, can be caused by several diseases, including idiopathic normal pressure hydrocephalus (INPH), and Parkinson’s disease (PD). We analyzed the relationship between these two conditions and their association with gait abnormalities using laboratory test data and findings from diffusion tensor imaging (DTI).

**Methods:**

The study involved 10 patients with INPH, 18 with PD, and 10 healthy individuals (control group). Fractional anisotropy (FA) of five brain areas was measured and compared among the three groups. In addition, the association of INPH and PD with gait capability, frontal lobe function, and FA of each brain area was evaluated.

**Results:**

The INPH group had significantly lower FA for anterior thalamic radiation (ATR) and forceps minor (Fmin) as compared to the PD group. The gait capability correlated with ATR FA in the INPH and PD groups. We found that adding DTI to the diagnosis assisted the differential diagnosis of INPH from PD, beyond what could be inferred from ventricular size alone.

**Conclusions:**

We expect that DTI will provide a useful tool to support the differential diagnosis of INPH and PD and their respective severities.

## Background

Idiopathic normal pressure hydrocephalus (INPH) is a syndrome which involves cognitive disorder, gait abnormalities, and urinary incontinence. INPH was first reported in 1965 by Hakim and Adams as a condition whose symptoms could be alleviated by a cerebrospinal fluid shunt [1]. In the past, patients with suspected INPH were frequently misdiagnosed. This over-diagnosis resulted in many unsuccessful surgeries or the development of postoperative complications and as a result, less attention was paid to INPH [2]. Under these conditions, three epidemiological studies were conducted on INPH, enabling estimation of its prevalence. The study by Hiraoka *et al*. found that 2.9% of community-dwelling elderly subjects showed radiological and clinical features consistent with INPH [3]. Using a similar procedure, Tanaka *et al*. reported possible INPH prevalence to be 1.4% [4] and Iseki *et al*. reported the prevalence of possible INPH to be 0.5%. Because symptoms appeared 4–8 years later in some of the initially symptom-free cases, the authors suggested the possibility that changes revealed by diagnostic imaging represent a precursor condition and proposed the concept “asymptomatic ventriculomegaly with features of idiopathic normal pressure hydrocephalus on MRI (AVIM)” [5]. These recent reports suggest the possibility that INPH is present in a latent form in a relatively high percentage of elderly people; thus, close attention should be paid to this disease concept.

Changes characteristic of INPH have also been reported from the analysis of head MRI, with the expression “disproportionately enlarged subarachnoid-space hydrocephalus (DESH).” DESH is presented as dilation of the ventricle accompanied by high convexity and narrowing of the median subarachnoid cavity [6-8]. To date, however, no clinically applicable technique of MRI evaluation enabling quantification of such a change has been established.

Disorders of the white matter are generally viewed as a pathological feature of INPH. In practice, pressure stimuli from the ventricle, ischemic demyelination, and micro infarction are visible in patients with INPH [9-11]. To evaluate INPH-associated changes in white matter *in vivo*, we adopted a diagnostic imaging technique, diffusion tensor imaging (DTI), recently introduced into clinical practice.

DTI enables physicians to collect overall information on diffusive motion (eigenvalue and eigenvector) through evaluation of the random motion of water molecules and their tensor analysis. Fractional anisotropy (FA), an indicator of anisotropy, is often used with DTI to provide a quantitative evaluation [12]. Basic research into diffusion anisotropy is currently under way, and it has been reported that the axonal membrane is a major factor which limits diffusion [13]. Therefore, the diffusion of water molecules in a voxel in white matter fibers aligned in a certain direction is more likely to be limited in the direction perpendicular to fiber alignment, resulting in intensification of diffusion anisotropy and FA elevation. Hence, DTI provides a useful tool for the detection of white matter degeneration. Analysis of FA with DTI has been used for the evaluation of changes in white matter associated with aging [14] and neurodegenerative diseases such as Parkinson’s disease (PD). Although reduction of FA in the genu of the corpus callosum, superior longitudinal fasciculus (SLF), cingulum, and substantia nigra has been reported for patients with PD, diverse methods have been used for the analysis of diffusion tensor and no consensus has yet been established regarding such changes [15].

Gait abnormalities in the elderly, characterized by shuffling, short steps and frozen gait, can be caused by several diseases, including INPH and PD. There is also a report that Parkinsonism develops from INPH [16]. Therefore, gait disturbance and Parkinsonism must be distinguished from INPH and Parkinsonian syndrome, including PD. Although both INPH and PD have a relatively high incidence among the elderly, no decisive method of early stage diagnosis has been established and many cases of these conditions appear to be left undetected [17-19]. Established methods of treatment are available for both diseases, and responses to shunt and drug therapy have been demonstrated. Therefore, the ability to distinguish between these two conditions is paramount.

The present study aimed to investigate the clinical applicability of DTI to analyze changes in the white matter of patients with INPH or PD using semi-automatic methods and a large number of regions of interest (ROIs).

## Methods

### Subjects

This study involved three groups of participants: patients with INPH (n = 10), patients with PD (n = 18), and healthy individuals (control group**,** n = 10). All participants were selected at the Nishinomiya Kyoritsu Neurosurgical Hospital. Diagnoses were made by neurosurgery and neurology specialists. Patients with focal cerebral vascular lesions such as cerebral infarction on T2-weighted MRIs and cardiovascular risk factors were excluded from the study. All 10 healthy individuals participated in the study for the purpose of comparison with the INPH and PD groups and did not show signs of intracranial lesions or neurological disease on T2-weighted MRIs and had no history of head trauma or psychiatric disease. The characteristics of each group are shown in Table 1. There was no statistically significant difference between any of the groups in terms of age or between the INPH and PD group for the “time to complete” in the Timed Up and Go (TUG) test for quantifying functional mobility [20] (one-way analysis of variance [ANOVA]). Each participant provided advanced written informed consent and the study was approved by the Institutional Review Board of the Nishinomiya Kyoritsu Neurosurgical Hospital, Japan.

**Table 1:** Profiles of the three patient groups

	**Control**	**INPH**	**PD**	**P-value**
N	10	10	18	
Age (years)	68.9 ± 2.2	73.9 ± 2.2	70.7 ± 1.6	0.263*
TUG, time to complete (s)	NA	21.2 ± 2.2	18.1 ± 1.6	0.253*
TUG, number of steps	NA	33.2 ± 3.3	24.4 ± 2.5	0.045* INPH > PD**
FAB, frontal assessment battery	NA	8.0 ± 0.8	14.7 ± 0.6	<0.001* INPH < PD**
MMSE, Mini mental state assessment	NA	19.0 ± 1.3	27.3 ± 1.0	<0.001* INPH < PD**
INPH diagnostic level (probable/definite)	NA	4/6	NA	
Hoehn-Yahr stage (range)	NA	NA	2.33 ± 0.2 (1–4)	
UPDRS unified PD rating scale motor score	NA	NA	20.8 ± 2.2	

*INPH*, idiopathic normal pressure hydrocephalus; *PD*, Parkinson’s disease; *TUG*, Timed Up and Go test; *FAB*, Frontal Assessment Battery; *MMSE*, Mini-Mental State Examination; *UPDRS*, Unified PD Rating Scale; *NA*, not assessed, Data represent mean ± SD,^*^One-way analysis of variance, ^**^Tukey’s post-hoc analysis.

### Patients with INPH

The diagnosis of INPH was made on the basis of symptoms, MRI studies, a lumbar puncture test, and surgical outcomes. INPH was diagnosed following the Japan INPH guidelines [21], which propose three levels of disease: possible, probable, and definite. Possible INPH requires meeting six criteria: (1) 1 or more of the clinical triad (gait disturbance, cognitive impairment, or urinary incontinence); (2) >60 years of age; (3) ventricular dilation (Evans Index, >0.3) with a narrow cerebrospinal fluid (CSF) space in the high convexity; (4) CSF pressure <200 mm H_2_O with a normal CSF protein level; (5) absence of another disease that could account for symptoms, and (6) no previous illness that could have caused ventricular dilation. Diagnosis of probable INPH includes the requirements for possible INPH and specifies that a patient with possible INPH shows an improvement in symptoms by a lumbar puncture test after removal of CSF. Patients with definite INPH must, in addition, show clinical improvement after CSF shunt surgery. In our study, the diagnosis of INPH was made when patients met the criteria of probable or definite INPH. Ten patients with probable or definite INPH were evaluated in this study. Motor and cognitive symptoms were evaluated by the TUG test, the Mini-Mental State Examination (MMSE) [22], and the Frontal Assessment Battery (FAB) test [23,24] in addition to MRI scans, prior to performing both a lumber puncture test and CSF shunt surgery.

### Patients with PD

Eighteen patients with PD were included in this study. The diagnosis of PD was made by a neurologist on the basis of the diagnostic criteria of the United Kingdom’s Parkinson’s Disease Society Brain Bank. The INPH and PD groups did not differ significantly with regard to age. We also collected information on gender, age at onset of disease, family history, severity of PD (Hoehn and Yahr [H&Y] stage) [25], motor score of Unified PD Rating Scale (UPDRS) [26], and gait and cognitive measurements as in INPH patients (TUG, MMSE, and FAB). This study excluded patients with severe immobility (H&Y stage 5) because of their inability to complete the TUG test, contraindications for MRI, and inability to give consent due to the presence of severely debilitating diseases. The characteristics of the patients with PD are shown in Table 1.

### DTI acquisition

MRI acquisition and analysis were performed with a methodology similar to those used by Koyama *et al*. [27]. DTI was performed using a 3-T MRI scanner (Trio; Siemens AG, Erlangen, Germany) with a 32-channel head coil. We used the standard fixed equipment to position the head so that it could scan in the section in alignment with AC-PC line (anterior commissure-posterior commissure line). Using a single-shot echo-planar imaging sequence, the DTI scheme included acquisition of 12 images with no collinear diffusion gradients (b = 1000 s/mm^2^) and 1 no diffusion-weighted image (b = 0 s/mm^2^). A total of 64 axial slices were obtained for each patient. The field of view was 230.4 × 230.4 mm^2^, the acquisition matrix was 128, and the slice thickness was 3 mm without a gap, resulting in voxel dimensions of 1.8 × 1.8 × 3.0 mm. The echo time was 83 ms, and the repetition time was 7000 ms. T1-weighted and T2-weighted MRIs were obtained for other diagnostic uses in addition to the DTI. The total time for MRI acquisition was approximately 20 min per patient. Patients unable to remain still, preventing complete MRI acquisition, were excluded from our database.

### Image processing

**Figure 1:**
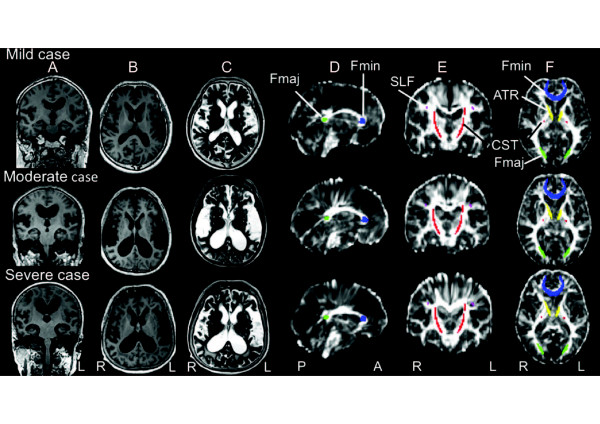
**Magnetic resonance images from three idiopathic normal pressure hydrocephalus (INPH) patients arranged in descending order of the anterior thalamic radiation fractional anisotropy (FA) level (mild, moderate and severe). A**) T1-weighted image (coronal view). **B**) T1-weighted image (axial view). **C**) T2-weighted image (axial view). **D**) Diffusion tensor image FA map (sagittal view). **E**) Diffusion tensor image FA map (coronal view). **F**) Diffusion tensor image FA map (axial view). Regions of interest (ROI) colors: forceps minor (Fmin, blue); anterior thalamic radiation (ATR, yellow); superior longitudinal fasciculus (SLF, purple); corticospinal tract (CST, red); forceps major (Fmaj, green).

**Figure 2:**
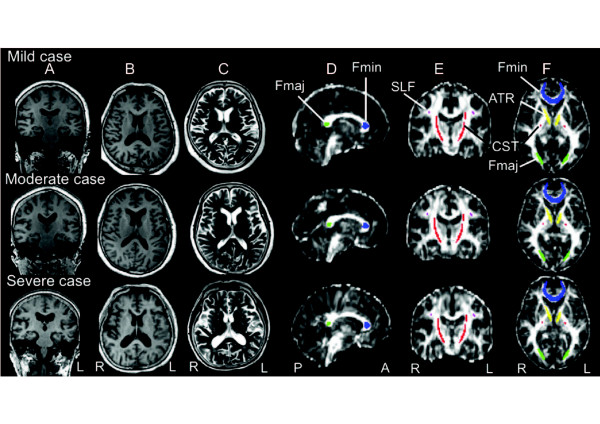
**Magnetic resonance images from three Parkinson’s disease (PD) patients in arranged descending order of anterior thalamic radiation fractional anisotropy (FA) level (mild, moderate and severe). A**) T1-weighted image (coronal view). **B**) T1-weighted image (axial view). **C**) T2-weighted image (axial view). **D**) Diffusion tensor image FA map (sagittal view). **E**) Diffusion tensor image FA map (coronal view). **F**) Diffusion tensor image FA map (axial view). Regions of interest (ROI) colors as for Figure 1. FA maps (FA values) of patients with PD (Figure 2D, E, F) show higher intensity (higher FA values) in white matter than those of patients with INPH (Figure 1 D, E, F).

The image processing was done in a blinded manner using the brain image analysis package FMRIB Software Library (FSL) [28], that comprised of various tools, including Brain Extraction Tool (BET), FMRIB’s Diffusion Toolbox (FDT), FMRIB’s Nonlinear Image Registration Tool (FNIRT), and FSLVIEW (interactive display tool for three-dimensional and four-dimensional data). Using the FDT tool to align all images in volumetric relation to the first image (b = 0 s/mm^2^), DTI data were corrected for motion and eddy current distortion. Next, extracerebral matter was excluded from the images using BET. DTI data were then analyzed using the FDT tool (FA brain map) to evaluate tensor diffusion and calculate each patient’s brain FA values. These FA values were mapped to a standard stereotaxic space (International Consortium of Brain Mapping DTI-81 Atlas: ICBM DTI-81 Atlas) using FNIRT [29,30]. To keep the transformation procedure simple, we employed “standard tasks” settings for FNIRT as recommended in the manual. Spatial transformations of the FA brain maps were confirmed by visual comparisons with images generated by FSLVIEW (Figures 1 and 2). When using FSLVIEW, we visually confirmed that the ROIs were within the white matter in all subjects (INPH, PD, and control groups).

Similar to previous studies [31-33], we specified an ROI in the forceps minor (Fmin), anterior thalamic radiation (ATR), superior longitudinal fasciculus (SLF), corticospinal tract (CST), and forceps major (Fmaj), using ROI templates as defined in the ICBM DTI-81 atlas (Figures 1 and 2). FA values were assessed using a code custom-written for the IDL software (ITTVIS, Osaka, Japan), and mean values for single voxels were then estimated.

### Statistical analyses

All statistical analyses were performed using the JMP software package (SAS Institute, Cary, NC,USA). FA values of the INPH, PD, and control groups were compared by ANOVA, followed by Tukey’s post-hoc analysis. We used Spearman’s rank correlation coefficient to identify possible associations between both the FA values of each ROI and the clinical symptoms (MMSE scores, FAB scores, TUG scores, H&Y stage, and UPDRS motor scores). Statistical significance was defined as *p < 0.05*.

To distinguish between INPH and PD, discriminant analysis was performed using the JMP software package. The discrimination correct rate was calculated by step-wise selection of 5 independent variables (TUG time to complete, TUG number of steps, MMSE scores, FAB scores, and FA values) (Pin = Pout = 0.05). Furthermore, the sensitivity and specificity in differentiating the INPH from PD were analyzed using Evans index (cut off 0.3). Receiver operating characteristic (ROC) curve analysis was performed to evaluate the diagnostic performance of the ATR FA values in differentiating the INPH from PD. We investigated which method of Evans index or ATR FA values was good for distinction of INPH and PD.

## Results

### DTI analyses of patients with INPH or PD

The INPH group had significantly lower FA for Fmin, ATR, SLF, CST, and Fmaj, as compared to the PD and control groups. The tendency for FA reduction in the INPH group was particularly noticeable in frontal lobe white matter ROIs, such as Fmin and ATR (*p* < 0.0001). There was no significant difference in FA between the PD and control groups for any variable measured (Table 2). We did not observe a significant correlation between the Evans index and FA for any of the five brain areas.

**Table 2:** Regions of interest (ROI) analysis of fractional anisotropy (FA) and Evans index for three groups of patients

**ROI**	**Control**	**INPH**	**PD**	**ANOVA**	**Tukey’s post-hoc**
Anterior thalamic radiation	0.426 ± 0.010	0.318 ± 0.010	0.417 ± 0.007	<0.0001	INPH < control = PD
Forceps minor	0.466 ± 0.011	0.375 ± 0.011	0.461 ± 0.008	<0.0001	INPH < control = PD
Superior longitudinal fasciculus	0.514 ± 0.020	0.421 ± 0.020	0.505 ± 0.015	0.0020	INPH < control = PD
Corticospinal tract	0.646 ± 0.009	0.608 ± 0.009	0.643 ± 0.006	0.0040	INPH < control = PD
Forceps major	0.762 ± 0.025	0.673 ± 0.025	0.750 ± 0.018	0.0252	INPH < control = PD
Evans index	0.28 ± 0.02	0.38 ± 0.04	0.29 ± 0.03	<0.0001	INPH > control = PD

INPH, idiopathic normal pressure hydrocephalus; PD, Parkinson’s disease; ANOVA, one-way analysis of variance;

Data represent mean ± SD (standard deviation). INPH < control = PD, significant FA reductions in INPH compared to control and PD, INPH > control = PD, significant Evans index elevation in INPH compared to control and PD.

**Figure 3:**
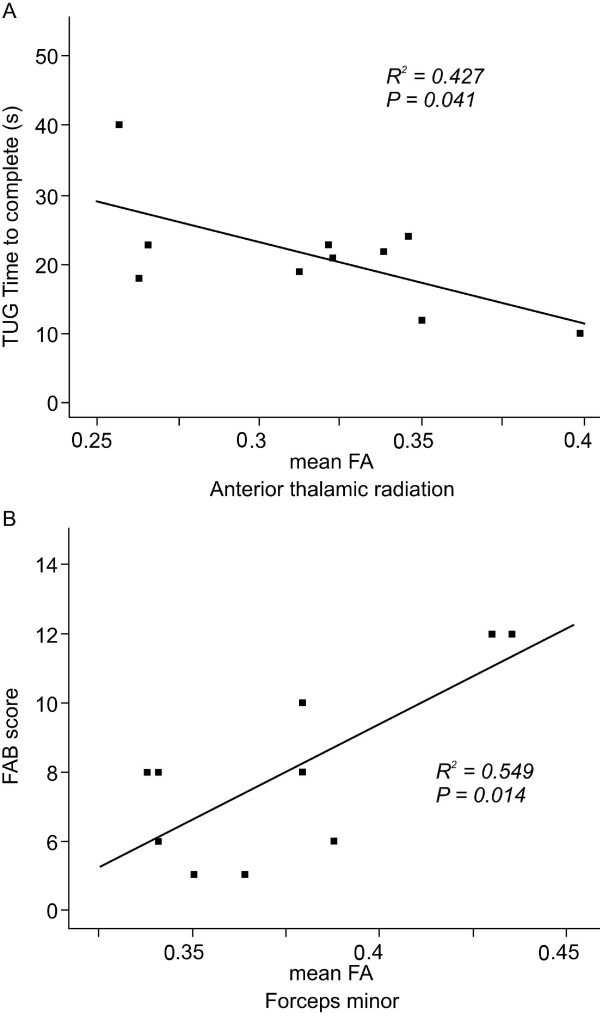
**Correlation graphs of the relationship between gait (A) and cognitive tests (B) and the mean fractional anisotropy (FA) in the idiopathic normal pressure hydrocephalus (INPH) group. A**) A negative correlation is seen between timed up and go (TUG) time to complete and FA for anterior thalamic radiation. **B**) A positive correlation is seen between frontal assessment battery (FAB) score and FA for forceps minor (Fmin).

In the INPH group, the TUG time correlated negatively with FA for ATR (*p* < 0.05**;** Figure 3A). In the same group, FAB scores correlated positively with FA for Fmin (*p* < 0.05; Figure 3B). However, neither TUG number of steps nor MMSE scores had a significant correlation with FA for any of the 5 brain areas.

**Figure 4:**
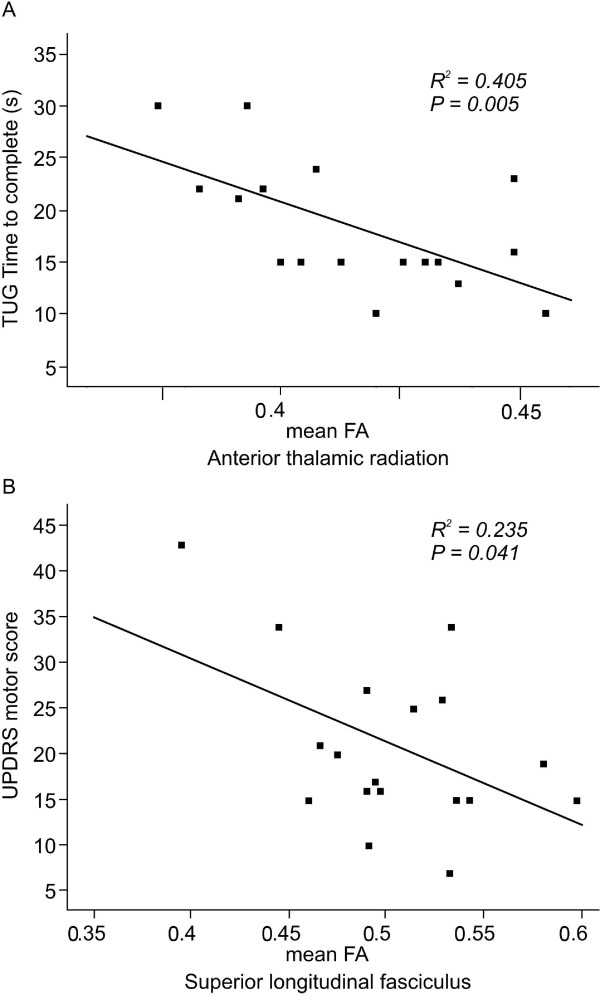
**Correlation graphs of the relationship between gait (A), and Parkinson’s disease (PD) rating scale (B) and the mean fractional anisotropy (FA) in the PD group. A**) A negative correlation is seen between the timed up and go (TUG) time to complete and FA for the anterior thalamic radiation. **B**) A negative correlation is seen between the unified PD rating scale (UPDRS) motor score and FA for superior longitudinal fasciculus.

In the PD group, the TUG time to complete correlated negatively with FA for ATR (*p* < 0.05; Figure 4A). In the same group, the UPDRS motor scores correlated negatively with FA for SLF (*p* < 0.05; Figure 4B). However, neither MMSE scores nor FAB scores had a significant correlation with FA for any of the 5 areas in the PD group.

### Discriminant analysis for the diagnosis

**Figure 5:**
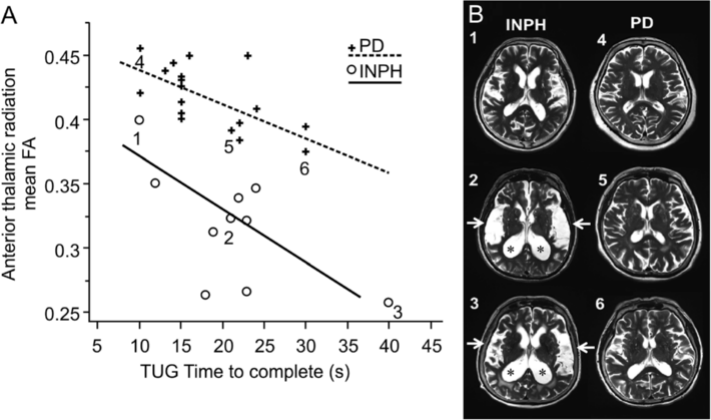
**Discriminant analysis for INPH and PD groups. A**) Association between fractional anisotropy (FA) for anterior thalamic radiation and TUG time to complete in the both the INPH and PD groups. A discriminant analysis indicates that distinction with a correct rate of 96.4% is possible on the basis of 2 independent variables (FA and TUG time to complete). This indicates that distinction between INPH and PD is possible on the basis of the finding that ATR FA is markedly lower in INPH patients than in PD patients, even when the TUG time to complete is similar. **B**) T2-weighted MRI images of the head are shown for cases 1, 2, and 3 from the INPH group and cases 4, 5, and 6 from the PD group in Figure 5A. The images of cases 2 and 3 with INPH show enlarged ventricles (*) and expanded Sylvian fissures (arrow)**.** When a comparison is made between early stage case 1 and case 4, the disproportionately enlarged subarachnoid space hydrocephalus (DESH) sign in case 1 is mild, and this makes the distinction between INPH and PD very difficult.

When discriminant analysis was carried out, the discrimination correct rate was 89.3% for the clinical assessment parameter TUG time to complete and FAB scores and 85.7% for TUG number of steps and FAB scores. Based upon this result, we attempted to improve the distinction rate by utilizing the data on FA values yielded from DTI. We specifically noted the finding that ATR FA was significantly lower in the INPH group than in the PD group, and that a negative correlation between TUG time to complete and ATR FA was present in both the INPH and PD groups. Regarding distinction between INPH and PD, the results of discriminant analysis suggested that distinction at a correct rate of 96.4% would be possible on the basis of ATR FA and TUG time to complete. It was demonstrated that distinction between INPH and PD is possible based upon the finding that ATR FA is markedly lower in INPH patients than in PD patients, even when the TUG time to complete is similar (Figure 5A). A similar tendency was also noted at the early stages of disease when symptoms were mild. Furthermore, the correct rate was improved to 100% when ATR FA, TUG time to complete, and FAB scores were selected by step-wise selection (Pin = Pout = 0.05) from the independent variables (ATR FA, TUG time to complete, TUG number of steps, MMSE scores, and FAB scores). These results indicate that evaluation of ATR’s white matter integrity by DTI is important, in addition to clinical evaluation of gait function and frontal lobe function, when a distinction must be made between INPH and PD. When an Evans index of 0.3 was used in differentiating the INPH from PD, the sensitivity was 1.0, and specificity was 0.78. We found large ventricles (Evans index >0.3) in four out of 18 patients with PD. When the ATR FA values were chosen as 0.35, the area under the ROC curve was greater than all other criteria, and the sensitivity and specificity for differentiating the two groups were 0.9, and 1.0, respectively.

## Discussion

### New findings from this study

This study suggests that changes in the white matter within the ATR affect the gait of patients with INPH or PD; however, the extent of these changes differed between INPH and PD (Figure 5A). FA was markedly reduced in patients with INPH, suggesting severe loss of white matter integrity. These results indicate that even when the extent of gait abnormalities is similar, ATR FA was lower in patients with INPH than in patients with PD. This relationship with regard to ATR FA appears to help distinguish between INPH and PD at the early clinical stages when the distinction between the conditions is particularly difficult (Figures 5A and B). Better specificity was shown when using the ATR FA values for differentiating INPH from PD as compared to Evans index. These results might apply to the subset of patients with Parkinsonism who also have large ventricles, and to patients at the early clinical stages.

The clinical application of diffusion tensor analysis with the semi-automatic methods reported by Koyama *et al*. [27] used in our study presents a novel attempt to use standard brain conversion by means of nonlinear registration and reproducible template ROIs for data analysis. In this way, analysis of FA in many brain regions was achieved easily and quickly.

### Comparison with previous studies

In the present study, the INPH group had significantly lower FA in all ROIs as compared to the PD group. FA reduction in the INPH group was particularly noticeable for frontal lobe white matter ROIs, such as Fmin and ATR. Furthermore, a negative correlation between TUG time to complete and ATR FA was noted in both the INPH and PD groups. In this context, Kanno *et al*. [33] reported that patients with INPH had a lower FA in the corpus callosum, SLF, sagittal stratum and anterior limb of the internal capsule, than patients with PD. They also found a negative correlation of TUG number of steps with subcortical white matter in the left supplementary motor area (SMA) and left anterior limb of the internal capsule. Although their study differs from our study in regards to ROI selection, their report supports our results regarding the presence or absence of distinction between right and left sides and parameters of TUG measured. Some investigators reported CST elevation with INPH as compared to the control group [31], but these discrepancies may be attributable to the presence or absence of registration of a standard brain, and differences in ROIs in CST. We speculated that in hydrocephalus, axonal degeneration was the major pathological feature [10]. White matter destruction is due to a combination of mechanical injury [34], impaired blood flow, and accumulation of waste products in the cerebrospinal fluid. Under the influence of anatomical spatial relationship, FA values of CST were increased due to compressed and stretched white matter. This happened for the ROI situated near the wall of the lateral ventricle [35]. Since ROI of CST used by this research was a comparatively long tract (it ranged from subcortical white matter to the brain stem), the influence of compression could have been reduced by the process of white matter destruction by other causes. Generally, in patients with INPH, FA values of many ROIs were decreased and there was a possibility that the same influence had taken place also in the CST in these experiments. In general, not all clinical symptoms of INPH were reversed by the shunt operation. This suggests that the cause of INPH included the reversible (e.g. compressed white matter) and irreversible (e.g. destruction of white matter) mechanisms. Furthermore, our study revealed a positive correlation between FAB scores and Fmin FA in the INPH group. This finding supports the validity of FAB (an often used conventional test on frontal lobe function) by means of tensor imaging.

FA in the frontal lobe [36], substantia nigra [37], and SLF [15] was reduced in patients with PD. We found that the PD group had no significant FA reduction in any of the five ROIs as compared to the control group. However, a negative correlation of FA with indicators of clinical seriousness (TUG time to complete and UPDRS motor score) was noted in the PD group. Taken together, these results suggest that the lack of FA reduction in the PD group in our study is attributable to the smaller number of PD patients who were at an earlier clinical stage of the disease as compared to PD patients in past studies.

### Common features of INPH and PD

Cortico-basal ganglia-thalamo-cortical circuits are commonly affected in patients with INPH or PD [38]. Parkinsonism features of INPH result from the loss of connectivity between the basal ganglia and the SMA [39]. Therefore, we took particular note of the negative correlation between TUG time to complete and ATR FA, which was present in both the INPH and PD groups, and we found that the INPH group showed significant reduction in FA. ATR is composed of white matter fibers projected from the thalamus to the frontal cortex, including the SMA, and other frontal areas that may play important roles in gait [40-42].

### Limitations

Limitations of this study include the fact that INPH and PD groups were comprised of patients capable of completing the TUG test, and the study involved comparison among cases at early clinical stages of the diseases. In addition, this was a relatively small-scale study, including 10 patients in the INPH group and 18 in the PD group. Further studies conducted with more patients would contribute much to the field.

Many neurologists would state that the specific gait abnormality in INPH looks different from PD, therefore the FA determination might merely support the diagnosis. However, INPH is a common disease which a non-specialist may also often encounter, and it seems that diagnosis by semi-automatic methods is very helpful.

In addition, the **c**oexistence of INPH with neurodegenerative diseases has been reported [43]. Therefore, it is possible that other neurodegenerative diseases, such as Lewy body dementia, Alzheimer’s disease or Progressive supranuclear palsy were present in the INPH group. Future studies are needed to examine this possibility.

## Conclusions

Diffusion tenor image analysis with semi-automatic methods is clinically applicable with respect to its reproducibility and simplicity. We expect that DTI will be a useful tool for assisting in the differential diagnosis of idiopathic normal pressure hydrocephalus and Parkinson’s disease and will help to assess function and disease stage in patients with these neurodegenerative diseases.

## Abbreviations

ATR: Anterior thalamic radiation; CST: Corticospinal tract; DESH: Disproportionately enlarged subarachnoid-space hydrocephalus; FA: Fractional anisotropy; FAB: Frontal assessment battery; Fmaj: Forceps major; Fmin: Forceps minor; DTI: Diffusion tensor imaging; H&Y stage: Hoehn & yahr stage; INPH: Idiopathic normal pressure hydrocephalus; MMSE: Mini mental state examination; MRI: Magnetic resonance imaging; PD: Parkinson’s disease; ROI: Region of interest; SLF: Superior longitudinal fasciculus; TUG: Timed up and go; UPDRS: Unified PD rating scale.

## Competing interests

The authors have no personal financial or institutional interest in any of the drugs, materials, or devices described in this article.

## Authors’ contributions

KM performed clinical evaluation, image processing and statistical analysis, participated in the study design and drafted the manuscript. TK conceived the study, participated in its design, coordinated the study between hospital departments, performed image acquisition and processing and helped to draft the manuscript. MH participated in the data interpretation. NK performed clinical evaluation and participated in data analysis. HM and KD participated in the design and organization of the study. All authors have read and approved the final version of the manuscript.
